# Dataset of biomass waste of rice paddies and forest sectors supporting the assessment of the potential for bioenergy production in Taiwan

**DOI:** 10.1016/j.dib.2019.104613

**Published:** 2019-10-04

**Authors:** Keng-Hao Chang, Kuo-Ren Lou, Chun-Han Ko

**Affiliations:** a Department of Management Sciences, Tamkang University, Taipei 25137, Taiwan; b School of Forest and Resources Conservation, National Taiwan University, Taipei, 10617, Taiwan

**Keywords:** Bioenergy, Biomass, Biomass waste, Harvested wood products (HWP), Rice paddy waste

## Abstract

Assessing the biomass-based bioenergy production potential of nations is heavily dependent on the import source of fossil fuels. Taiwan can alleviate its fossil fuel dependency and greenhouse gas emissions. Key information (including published statistics and calculations) on biomass waste production from rice paddies in Taiwan considering the full production of available rice paddies was included in this study. In addition, key information on biomass waste production from forest sectors in Taiwan considering enhanced domestic production and the imported sources of harvested wood products (HWP) was also included. The volumes of decayed HWP could be used to predict available biomass waste for bioenergy production. The results of this study assist with the recent evaluation of the potential of the most prevailing available sources of biomass waste, rice paddies and forest sectors, for bioenergy generation, e.g. for more insight please see “Potential of Bioenergy Production from Biomass Wastes of Rice Paddies and Forest Sectors in Taiwan” [1].

**Table:** Specifications Table

Subject area	*Renewable Energy, Sustainability and the Environment*
More specific subject area	*Biomass and bioenergy.*
Type of data	*Text, tables.*
How data were acquired	*Raw, complied from government published dataset, etc.*
Data format	*Raw, analyzed, calculated, etc.*
Data source location	*Taipei, Taiwan, ROC.*
Data accessibility	[1] Council of Agriculture, 2017. Accounts of green national income: agricultural solid wastes (In Chinese). http://agrstat.coa.gov.tw/sdweb/public/common/Download_file.ashx?list_id=18 (accessed May 24, 2018) Council of Agriculture, Taiwan, ROC. [2] Forestry Bureau, C., Executive Yuan, ROC, 2017. Forestry Statistics 2016 Yearbook. Forestry Bureau, Council of Agriculture, Taiwan, ROC. https://www.forest.gov.tw/EN/0001465 (accessed May 24, 2018) [3] MOEADOS, 2018. Industrial statistics-industrial production, shipment and inventory survey (In English). Department of Statistics, Ministry of Economic Affairs, R.O.C. http://dmz9.moea.gov.tw/gmweb/advance/AdvanceQuery.aspx (accessed May 24, 2018). [4] Customs Administration, 2017. Query system for statistics database (In Chinese). Customs Administration, Ministry of Finance, R.O.C. https://portal.sw.nat.gov.tw/APGA/GA03 (accessed May 24, 2018) Author's name Title Journal DOI
Related research article	Author's name: Chang, K. H., Lou, K.R., Ko, C.H. Title: Potential of Bioenergy Production from Biomass Wastes of Rice Paddies and Forest Sectors in Taiwan. Journal: Journal of Cleaner Production DOI: https://doi.org/10.1016/j.jclepro.2018.09.048

**Table:** 

**Value of the Data** • The dataset contains key information pertaining to biomass waste production from rice paddies in Taiwan considering the full production of available rice paddies. • The dataset contains key information pertaining to biomass waste production from forest sectors in Taiwan considering enhanced domestic production and associated waste from harvested wood products. • The dataset can be used to estimate the potential for bioenergy production in Taiwan from post-consumer biomass from all kinds of harvested wood products.

## 1 Data

The data presented in this article are employed to facilitate the analysis and prediction of the research article “Potential of Bioenergy Production from Biomass Wastes of Rice Paddies and Forest Sectors in Taiwan” [1] from 2017 to 2065. Governmental statistic data of this article are from 1990. Governmental statistics for rice straw and rough rice production [2], listed by Table 1, were employed to estimate biomass wastes of rice paddies.

**Table 1:** Historical data on rice husks and rice straw in Taiwan [2].

Year	Original data		Converted values	
	Rice husks (tons)	Rice Straw (tons)	Rice husks (T g y^−1^)	Rice Straw (T g y^−1^)
1990	456,734	2,283,670	0.45673	2.28367
1991	462,328	2,311,638	0.46233	2.31164
1992	413,976	2,069,880	0.41398	2.06988
1993	446,587	2,232,933	0.44659	2.23293
1994	412,281	2,061,403	0.41228	2.06140
1995	414,394	2,071,968	0.41439	2.07197
1996	386,179	1,930,897	0.38618	1.93090
1997	408,369	2,041,843	0.40837	2.04184
1998	371,831	1,859,157	0.37183	1.85916
1999	383,261	1,916,305	0.38326	1.91630
2000	381,211	1,906,057	0.38121	1.90606
2001	349,000	1,745,000	0.34900	1.74500
2002	365,000	1,825,000	0.36500	1.82500
2003	329,000	1,648,000	0.32900	1.64800
2004	286,722	1,433,611	0.28672	1.43361
2005	293,428	1,467,139	0.29343	1.46714
2006	311,609	1,558,048	0.31161	1.55805
2007	272,691	1,363,458	0.27269	1.36346
2008	291,435	1,457,175	0.29144	1.45718
2009	315,634	1,578,169	0.31563	1.57817
2010	290,201	1,451,011	0.29020	1.45101
2011	333,255	1,666,237	0.33326	1.66624
2012	340,046	1,700,229	0.34005	1.70023
2013	317,912	1,589,564	0.31791	1.58956
2014	346,442	1,732,210	0.34649	1.73245
2015	316,346	1,581,732	0.31637	1.58186
2016	332,759	1,663,803	0.33275	1.63380

Table 2 lists historical data of domestic logging volumes and estimation of biomass waste from domestic logging in Taiwan [3]. Table 3 lists the tax codes of Customs Administration for each respective item of wood products [4] for further calculation and prediction of harvested wood products (HWP) contribution of biomass waste. Table 4 lists statistics on domestically produced HWP from 1990 to 2016. The first data column of Table 4, sawn timber and firewood production, is compiled from Forestry Bureau [3]. Data from other later columns for statistics for domestically produced HWP are of other items published from the Ministry of Economic Affairs [5].

**Table 2:** Historical domestic logging volumes and estimation of biomass waste from domestic logging in Taiwan [3].

Year	Data	Logging slash			Log-processing waste	
	Domestic logging volume (m^3^)	Above ground biomass volume (m^3^)	Slash volume (m^3^)	Slash mass (tons)	Waste volume (m^3^)	Waste mass (tons)
1990	203,313	221,232	17,920	8960	19,286	9643
1991	126,059	137,170	11,111	5555	11,958	5979
1992	118,323	128,752	10,429	5214	11,224	5612
1993	71,735	78,057	6323	3161	6805	3402
1994	56,128	61,075	4947	2474	5324	2662
1995	63,177	68,745	5568	2784	5993	2996
1996	56,362	61,329	4968	2484	5346	2673
1997	52,173	56,772	4598	2299	4949	2475
1998	49,529	53,894	4365	2183	4698	2349
1999	42,945	46,730	3785	1893	4074	2037
2000	35,179	38,280	3101	1550	3337	1669
2001	47,859	52,077	4218	2109	4540	2270
2002	61,060	66,441	5382	2691	5792	2896
2003	85,542	93,082	7540	3770	8115	4057
2004	70,801	77,042	6240	3120	6716	3358
2005	60,058	65,352	5293	2647	5697	2849
2006	63,596	69,201	5605	2803	6033	3016
2007	67,219	73,143	5925	2962	6376	3188
2008	51,108	55,612	4505	2252	4848	2424
2009	44,281	48,184	3903	1951	4200	2100
2010	32,799	35,690	2891	1445	3111	1556
2011	36,913	40,167	3253	1627	3502	1751
2012	46,230	50,305	4075	2037	4385	2193
2013	42,219	45,940	3721	1861	4005	2002
2014	62,271	67,760	5488	2744	5907	2954
2015	51,608	56,157	4549	2274	4896	2448

**Table 3:** HWP tax codes for regarding items from the Customs Administration [6].

Categories	Item tax codes	Description
Sawn wood		
	4403	Rough wood; whether stripped of bark or sapwood, or roughly squared
	4407	Wood sawn, chipped lengthwise, sliced or peeled; whether planed, sanded or end-jointed, of a thickness exceeding 6 mm
Wood panel		
	4408	Sheets for veneering (including those obtained by slicing laminated wood), for plywood or for similar laminated wood and other wood, sawn lengthwise, sliced or peeled; whether planed, sanded, spliced or end-jointed, of a thickness not exceeding 6 mm
	4410	Particle board, oriented strand board (OSB) or similar boards (for example, waferboard) of wood or other ligneous materials; whether agglomerated with resins or other organic binding substances
	4411	Fiberboard of wood or other ligneous materials; whether bonded with resins or other organic substances
	4412	Plywood, veneered panels and similar laminated wood
Paper		
	48	Paper and paperboard; articles of paper pulp, paper or paperboard
	47	Wood pulp or pulp of other fibrous cellulosic material; (waste and scrap) paper or paperboard

**Table 4:** Statistics on domestically produced HWP from 1990 to 2016 [3,5].

Year	Sawn wood		Wood panel		Paper				
	Saw timber (m^3^)	Lumber (m^3^)	Plain plywood (10^3^ m^2^)	Fancy plywood (10^3^ m^2^)	Fine paper (tons)	Package paper (tons)	Chinese art paper (tons)	Paper board (tons)	Family and Hygiene paper (tons)
1990	203313	697373	126065	114901	583528	86135	9779	2,508,788	140741
1991	126059	757833	173416	105325	629345	85436	9078	2,855,974	163014
1992	118323	564999	158181	96765	679043	93427	7440	2,950,366	177826
1993	71735	398796	113249	86064	695147	92845	7558	2,836,623	154392
1994	56128	378108	83278	77338	771551	94967	8535	3,045,500	156261
1995	63177	328537	64960	78026	792263	95332	8038	3,030,337	161045
1996	56362	366836	48870	68763	694944	86891	7728	3,213,194	176377
1997	52173	278747	46991	73463	805098	92776	8719	3,253,776	187861
1998	49529	270344	41811	64585	812263	89077	9212	2,975,404	186123
1999	42945	271862	39457	56701	821459	83415	8943	3,073,545	188229
2000	35179	232880	29488	51168	796916	82395	9396	3,233,864	217713
2001	47859	214399	29285	40672	722100	73930	7526	2,590,530	191281
2002	61060	204224	27366	36330	757692	80553	7349	3,235,255	191159
2003	85542	199547	20687	35030	740323	86959	7736	3,403,838	199696
2004	70801	239048	19748	44089	737370	91799	8010	3,554,986	217862
2005	60058	180992	14435	45651	748966	91111	6868	3,377,999	216637
2006	63596	191160	13105	46928	761108	94914	6941	3,350,646	206973
2007	67219	135334	14477	45869	777396	94589	6188	3,406,476	198974
2008	51108	135731	12978	42736	704294	84351	6397	2,909,620	206163
2009	44281	73641	10175	36258	692095	63700	5178	2,775,312	189711
2010	32799	78599	9344	40280	699910	63726	6553	2,850,379	197901
2011	36913	81017	12582	41029	690832	50992	6143	2,931,064	205255
2012	46230	88179	12169	40306	671309	46556	6207	3,073,574	201420
2013	42219	87224	12799	42365	619554	47223	6265	3,054,693	205710
2014	62271	96408	10053	41916	620916	44326	6915	3,083,250	206327
2015	51608	73039	8667	40383	531247	43606	6750	2,858,981	209099
2016	51608	72274	8834	40159	460924	39022	6425	3,009,632	218246

Table 5 lists imported HWP data from the Customs Administration [4]; tonnage data of subcategory items of wood panels were converted from square meters and thickness of plywood. Table 6 lists the exported HWP data from all respective items listed and published by Customs Administration [4]. Table 7 lists overall biomass tonnage of imported and exported HWP data, derived from the summation of data from Table 5, Table 6. Table 8 lists carbon mass overall biomass tonnage of imported and exported HWP data, using IPCC methodology [6] and derived from summation of data from Table 5, Table 6. Table 9 lists carbon inflow statistics of HWP from 2007 to 2016, summarizing data from Table 7, Table 8. Carbon inflow statistics of HWP serve as the base for estimating biomass waste of forest sectors by IPCC method [6] for future scenario [1].

**Table 5:** Statistics on imported HWP [4].

Year	Sawn wood		Wood panels				Paper	
	4403 (tons)	4407 (tons)	4408 (tons)	4410 (tons)	4411 (tons)	4412 (tons)	48 (tons)	47 (tons)
1990	3,556,219	481,151	63,968	79,631	180,753	229,208	537,802	1,873,785
1991	3,771,429	652,576	104,532	102,245	238,752	290,977	638,988	2,568,476
1992	3,620,151	969,972	175,656	113,602	233,351	481,228	847,492	2,347,449
1993	1,814,673	1,245,246	170,848	158,276	263,617	447,477	975,232	2,359,436
1994	1,830,331	1,009,816	174,453	123,010	323,974	663,476	1,227,066	2,709,740
1995	1,542,206	985,975	121,578	138,794	211,887	597,852	1,158,153	2,200,618
1996	1,559,635	792,797	119,183	144,000	187,863	485,991	1,056,645	2,567,599
1997	1,605,037	880,320	159,368	188,489	248,534	542,954	1,336,283	2,341,199
1998	1,063,796	732,324	139,187	178,519	196,560	552,880	1,269,896	1,949,822
1999	1,118,739	742,837	133,834	199,694	255,358	379,600	1,400,343	2,108,077
2000	1,020,521	757,472	119,910	216,411	229,358	433,675	1,370,377	1,984,712
2001	826,384	571,247	107,712	170,919	164,006	325,888	1,199,988	1,941,914
2002	774,477	666,977	107,798	197,229	196,985	379,908	1,278,073	1,921,250
2003	835,587	686,490	105,102	182,013	183,121	429,372	1,476,053	2,032,646
2004	912,427	818,671	114,455	219,389	177,346	528,806	1,645,072	1,883,577
2005	870,884	785,159	98,764	177,950	177,858	544,442	1,581,012	1,661,349
2006	721,641	757,401	101,327	187,580	147,018	567,086	1,503,597	1,799,765
2007	756,713	723,355	99,242	189,787	140,069	503,345	1,431,735	1,960,407
2008	631,877	676,545	110,608	168,690	120,317	417,944	1,467,889	1,707,298
2009	523,491	494,701	89,454	162,432	97,257	323,537	1,265,690	1,407,702
2010	667,745	716,283	130,130	201,281	129,144	447,512	1,491,583	1,411,490
2011	584,231	769,962	133,065	208,171	115,156	404,887	1,535,667	1,458,062
2012	619,817	691,683	135,395	112,029	112,029	382,583	1,530,476	1,707,045
2013	580,098	732,897	150,192	189,670	107,829	406,959	1,514,904	1,619,608
2014	632,834	808,224	136,914	227,738	115,598	417,460	1,495,379	1,561,077
2015	479,955	763,998	149,165	237,286	101,283	375,603	1,446,283	1,355,400
2016	458,523	677,629	128,663	218,112	99,494	349,837	1,472,751	1,556,266

**Table 6:** Statistics of exported HWP [6].

Year	Sawn wood		Wood panels				Paper	
	4403 (tons)	4407 (tons)	4408 (tons)	4410 (tons)	4411 (tons)	4412 (tons)	48 (tons)	47 (tons)
1990	9096	18,927	3413	1895	6287	134,079	612,712	111,323
1991	4688	18,314	2777	2195	1594	107,006	852,855	80,512
1992	19,007	17,185	1809	4201	1919	89,836	832,377	48,554
1993	11,951	19,604	2999	5296	5330	64,199	790,445	37,773
1994	6768	22,467	2581	6447	8010	74,498	896,662	9366
1995	5644	25,343	5822	3987	7156	86,522	959,679	15,363
1996	10,304	25,773	2739	1451	5262	92,507	1,186,710	3681
1997	7286	32,894	1946	1204	4182	86,734	1,087,368	5879
1998	5987	28,724	4118	2335	6483	45,753	987,900	2753
1999	8259	31,145	2516	2386	6969	40,729	1,024,236	16,670
2000	11,965	33,512	3699	2372	3932	40,762	1,090,498	23,391
2001	15,292	35,151	3909	2509	5149	33,801	1,189,204	25,931
2002	14,480	32,847	5096	3052	10,060	27,855	1,343,071	26,647
2003	10,155	37,585	6243	2275	11,351	21,052	1,347,286	37,911
2004	18,020	44,513	6983	3252	14,524	19,271	1,505,404	37,025
2005	26,239	38,033	9512	4283	8650	16,562	1,506,027	49,271
2006	23,949	33,862	8349	2063	10,665	19,008	1,585,687	69,106
2007	35,717	30,074	6643	1471	8701	18,388	1,699,496	84,228
2008	27,427	25,908	5908	1145	8656	22,080	1,360,286	35,030
2009	25,663	22,365	3948	1112	8906	18,005	1,459,544	58,325
2010	16,013	22,110	3096	782	6439	22,112	1,325,669	106,133
2011	21,773	19,688	3268	1175	4981	20,492	1,388,239	93,054
2012	31,379	20,665	3102	5431	5431	17,920	1,545,667	100,755
2013	44,009	22,586	2899	1994	5993	22,371	1,526,617	102,788
2014	40,516	18,421	3212	2233	5395	17,666	1,474,061	124,689
2015	23,587	13,806	2427	2624	5229	11,987	1,196,388	171,896
2016	22,641	13,340	2130	2534	4109	10,347	1,376,178	254,201

**Table 7:** Biomass tonnage of imported and exported HWP.

Year	Import			Export		
	Sawn wood (tons)	Wood panels (tons)	Paper (tons)	Sawn wood (tons)	Wood panels (tons)	Paper (tons)
1990	3,633,633	498,205	2,170,429	25,220	131,107	651,632
1991	3,981,605	662,856	2,886,718	20,702	102,215	840,030
1992	4,131,111	903,454	2,875,447	32,573	87,988	792,838
1993	2,753,928	936,196	3,001,202	28,400	70,042	745,396
1994	2,556,133	1,156,422	3,543,125	26,312	82,383	815,425
1995	2,275,363	963,101	3,022,894	27,888	93,138	877,538
1996	2,117,189	843,333	3,261,819	32,470	91,763	1,071,352
1997	2,236,822	1,025,411	3,309,734	36,162	84,659	983,923
1998	1,616,508	960,432	2,897,746	31,239	52,819	891,588
1999	1,675,418	871,638	3,157,577	35,464	47,339	936,815
2000	1,600,193	899,419	3,019,580	40,930	45,688	1,002,500
2001	1,257,868	691,672	2,827,712	45,398	40,831	1,093,621
2002	1,297,309	793,728	2,879,391	42,594	41,457	1,232,746
2003	1,369,869	809,648	3,157,829	42,966	36,829	1,246,677
2004	1,557,988	935,996	3,175,784	56,279	39,627	1,388,186
2005	1,490,439	899,112	2,918,125	57,845	35,107	1,399,768
2006	1,331,138	902,710	2,973,025	52,030	36,076	1,489,314
2007	1,332,061	839,199	3,052,928	59,212	31,683	1,605,351
2008	1,177,580	735,803	2,857,668	48,002	34,010	1,255,785
2009	916,372	605,412	2,406,053	43,226	28,774	1,366,082
2010	1,245,625	817,261	2,612,766	34,311	29,186	1,288,622
2011	1,218,774	775,152	2,694,356	37,315	26,924	1,333,164
2012	1,180,350	667,832	2,913,769	46,840	28,696	1,481,780
2013	1,181,696	769,185	2,821,061	59,935	29,931	1,466,464
2014	1,296,952	807,938	2,750,810	53,044	25,656	1,438,875
2015	1,119,557	777,004	2,521,515	33,653	20,041	1,231,456
2016	1,022,537	716,496	2,726,115	32,383	17,209	1,467,341

**Table 8:** Carbon conversion from HWP statistics.

Item	Volume or mass			Converted carbon mass		
	Sawn wood	Wood panel	paper	Sawn wood	Wood panel	Paper
Year	m^3^	m^3^	ton	C ton	C ton	C ton
1990	900,686	765,067	3,328,971	202,654	224,930	1,498,037
1991	883,892	885,003	3,742,847	198,876	260,191	1,684,281
1992	683,322	809,454	3,908,102	153,747	237,979	1,758,646
1993	470,531	632,819	3,786,565	105,869	186,049	1,703,954
1994	434,236	509,956	4,076,814	97,703	149,927	1,834,566
1995	391,714	453,981	4,087,015	88,136	133,470	1,839,157
1996	423,198	373,485	4,179,134	95,219	109,805	1,880,610
1997	330,920	382,441	4,348,230	74,457	112,438	1,956,704
1998	319,873	337,807	4,072,079	71,971	99,315	1,832,436
1999	314,807	305,302	4,175,591	70,832	89,759	1,879,016
2000	268,059	256,083	4,340,284	60,313	75,288	1,953,128
2001	262,258	222,113	3,585,367	59,008	65,301	1,613,415
2002	265,284	202,235	4,272,008	59,689	59,457	1,922,404
2003	285,089	176,901	4,438,552	64,145	52,009	1,997,348
2004	309,849	202,682	4,610,027	69,716	59,589	2,074,512
2005	241,050	190,773	4,441,581	54,236	56,087	1,998,711
2006	254,756	190,605	4,420,582	57,320	56,038	1,989,262
2007	202,553	191,599	4,483,623	45,574	56,330	2,017,630
2008	186,839	176,892	3,910,825	42,039	52,006	1,759,871
2009	117,922	147,425	3,725,996	26,532	43,343	1,676,698
2010	111,398	157,556	3,818,469	25,065	46,322	1,718,311
2011	117,930	170,215	3,884,286	26,534	50,043	1,747,929
2012	134,409	166,608	3,999,066	30,242	48,983	1,799,580
2013	129,443	175,146	3,933,445	29,125	51,493	1,770,050
2014	158,679	165,002	3,961,734	35,703	48,510	1,782,780
2015	124,647	155,734	3,649,683	28,046	45,786	1,642,357
2016	123,882	155,553	3,734,249	27,873	45,733	1,680,412
2017	69,116	156,931	3,827,140	15,551	46,138	1,722,213

**Table 9:** Carbon inflow statistics on HWP from 2007 to 2016.

	Sawn wood (metric tons C)				Wood panel (metric tons C)				Paper (metric tons C)					
	Import	Export	Domestic production	Inflow	Import	Export	Domestic production	Inflow	Import	Export	Domestic production		Inflow	
2007	666,031	29,606	45,574	681,999	392,745	14,828	56,330	434,248	1,526,464	802,676	2,017,630		2,741,418	
2008	588,790	24,001	42,039	606,828	344,356	15,917	52,006	380,445	1,428,834	627,892	1,759,871		2,560,813	
2009	458,186	21,613	26,532	463,106	283,333	13,466	43,343	313,209	1,203,026	683,041	1,676,698		2,196,683	
2010	622,812	17,155	25,065	630,721	382,478	13,659	46,322	415,141	1,306,383	644,311	1,718,311		2,380,383	
2011	609,387	18,658	26,534	617,263	362,771	12,600	50,043	400,214	1,347,178	666,582	1,747,929		2,428,525	
2012	590,175	23,420	30,242	596,997	312,545	13,430	48,983	348,098	1,456,885	740,890	1,799,580		2,515,575	
2013	590,848	29,968	29,125	590,005	359,979	14,008	51,493	397,464	1,410,530	733,232	1,770,050		2,447,348	
2014	648,476	26,522	35,703	657,657	378,115	12,007	48,510	414,618	1,375,405	719,438	1,782,780		2,438,748	
2015	559,779	16,827	28,046	570,998	363,638	9379	45,786	400,044	1,260,757	615,728	1,642,357		2,287,387	
2016	511,269	16,192	27,873	522,951	335,320	8054	45,733	372,999	1,363,058	733,670	1,680,412		2,309,799	
Avg.		22,396		593,853		12,735		387,648		696,746			2,430,668	

## 2 Experimental design, materials, and methods

### 2.1 Biomass waste from rice paddy

Governmental statistics for rice straw and rough rice production [2], listed by Table 1, were employed to estimate biomass wastes of rice paddies. Historical and converted data on rice husks and rice straw in Taiwan [2] are listed in Table 1. Original data are to the oven dried values converted from published data [2], where rice husks and rice straw are respectively 20% and equal weight of produced rice. Converted values, as listed in row 3 and 4, were the conversion of original data of the first two rows of air dried weights to weights expressed of SI units.

### 2.2 Biomass waste from domestic logging

Table 2 lists historical data of domestic logging volumes and estimation of biomass waste from domestic logging in Taiwan [3]. Published domestic logging volumes were directly taken from the statistics provided from the Forestry Bureau [3]. Logging slash is the deduction of logging volume from the above ground biomass volume estimated by the IPCC method [6]. Of Taiwan plantation forests distribution, there are 37% softwood plantation forests, 42% of hardwood plantation forests and 21% mixed softwood and hardwood plantation forests [3]. Biological expansion factors (BEF) of softwood, hardwood and 21% mixed softwood and hardwood plantation are 1.27, 1.40 and 1.34, respectively [7].

Above ground biomass volume = (Domestic logging volume * 1.27) ÷ (1 + 0.22) * 0.37 + (Domestic logging volume * 1.40)÷(1 + 0.24) * 0.42 + (Domestic logging volume * 1.34) ÷ (1 + 0.23) * 0.21.

Wood density is 0.5 ton/m^3^ to convert slash volume to slash mass. Domestic survey conducted by Chen et al. [8] indicates that ratios for log-processing waste for sawn wood and wood panel are 36.3 and 28.5%.

### 2.3 Biomass and carbon inflow from HWP

Based on the IPCC definition [4], there are three categories of HWP: sawn wood, wood panel and paper products. Each category of HWP contains a variety of respective products, listed as item, on the current tax code system of the Customs Administration [4]. Table 3 lists the tax codes of each respective item of HWP Customs Administration [4] for further calculation and prediction of HWP contribution of biomass waste in 2017–2065 [1]. Governmental statistics of each item of HWP from customs administration is employed to further calculate and predict biomass wastes derived from imported and exported HWP.

Biomass waste from forest sectors consisted of waste from domestically harvested wood product (HWP), from available processing waste from domestic harvest and the production of HWP, as well as from domestically produced and imported HWP. Table 4 lists statistics on domestically produced HWP from 1990 to 2016. The data first column of Table 4, sawn timber production, is from Forestry Bureau [3]. Data from other columns for statistics for domestically produced HWP are data of other items published from the Ministry of Economic Affairs [7]. According to the last 10-year average from survey by the Ministry of Economy [5], the volumetric ratios of wood panels, sawn wood, and paper consumed in Taiwan were 12, 18, and 70%, respectively.

Table 5 lists imported HWP data from the Customs Administration [6]; tonnage data of subcategory items of wood panels were converted from square meters and thickness of plywood. The thickness of both plain and fancy plywood, 3.175 mm, is from a domestic survey [8].

Table 6 lists exported HWP data from all respective items listed and published by Customs Administration [6].

Table 7 lists overall biomass tonnage of imported and exported HWP data, derived from summation of data from Table 5, Table 6. Statistics on sawn wood are obtained by adding items 4403 and 4407. The ratio of oven to air dried masses of sawn wood is 0.9. Statistics on wood panels are obtained by adding items 4408, 4410, 4411 and 4412. Statistics on paper are obtained by adding items 47 and 48. The ratio of oven to air dried masses of paper is 0.9 [6].

Table 8 lists carbon mass overall biomass tonnage of imported and exported HWP data, using IPCC methodology [6] and derived from summation of data from Table 5, Table 6. Carbon ratio to overall dried mass for sawn wood, wood panels and paper are 0.225, 0.294 and 0.45 C ton/m^3^, respectively [6].

Table 9 lists carbon inflow statistics on HWP from 2007 to 2016, summarizing data from Table 7, Table 8. This study assumes that ratios of the post-consumer HWP in the study period of [1] remain constant. The results of this study facilitate the evaluation of the potential for bioenergy generation in Taiwan from available biomass waste from the two most prevailing sources, rice paddies and forest sectors. Carbon inflow values of each category of forest sector include the import plus domestic production deduced by export values.
